# The ROCK Tool: A Novel Method for the Structural Exploration of Schemata

**DOI:** 10.3389/fpsyg.2021.675938

**Published:** 2021-07-13

**Authors:** Bohao Shi, Zhenhui Jiang, Jifan Zhou, Hui Chen

**Affiliations:** Department of Psychology and Behavioral Sciences, Zhejiang University, Zhejiang, China

**Keywords:** serial reproduction paradigm, Big Five theory, hierarchical clustering, iteration, Markov chain

## Abstract

Information stored in the human memory is organized in the form of mental schemata. In this paper we report on the Reproduction of Categorical Knowledge (ROCK) tool, a novel method for uncovering the structure of mental schemata of memorized information. The tool applies serial reproduction and hierarchical clustering to magnify memory bias and uncover inner configurations of fragmented information, using strength of association. We conducted behavioral experiments to test the validity of the tool. Experiment 1a demonstrated that the schematic structure of personality traits uncovered by the ROCK tool highly matched those described by the Big Five theory. This finding was replicated in Experiment 1b, focusing on a lower-level personality dimension extroversion with results aligned with personality theories. Experiment 2 assessed the ROCK tool using artificial stimuli with a pre-defined structure, created using a Markov chain model. Participants acquired the structure of the stimuli through an implicit learning procedure, and the ROCK tool was used to assess their level of recall. The results showed that the learned structure was identical to the designed structure of the stimuli. The results from both studies suggest that the ROCK tool could effectively reveal the structure of mental schemata.

## Introduction

Memory is affected by knowledge and prior experience (Bartlett, 1932), and provides a means to explore people’s representation of ideas and opinions. Memory is regarded as the active organization of past reactions (De Brigard et al., 2017), so that each time new information is encoded into previously related events, an integrated knowledge structure will be generated for later memory retrieval. This knowledge structure is called a schema (Piaget, 1923; Dimaggio, 1997). More specific proposals of schematic concepts include script accounts for situational knowledge (Schank and Abelson, 1977), and frame, which is a remembered framework for adopting details to fit reality (Minsky, 1988).

The effects of schemata on memory occurs after encoding and later during retrieval (Stein and Glenn, 1975; Brewer and Treyens, 1981; Poldrack et al., 2001; Roediger and McDermott, 1995; Ramponi et al., 2004). Memory advantages of schema-relevant, as compared to schema-irrelevant information, have been found for both recall and recognition. Firstly, relative to schema-irrelevant information, schema-relevant information leads to better free recall performance for story content or scenario actions (McDaniel, 1984; Gopher et al., 1985; Yussen et al., 1988; Geiselman and Callot, 1990), as well as better cued recall performance for schema-relevant information in reconstructing relations or types of variables from memorized algebra problems and word pairs association (Roediger and Adelson, 1980; Mayer, 1982; Schoenfeld and Herrmann, 1982). It has been suggested that this is the result of the strong connection between schema-relevant information and its context (Cann, 1993; Ginet et al., 2013). Dijksterhuis and Van Knippenberg (1995) proposed that when recalling impression-related information, in order to form an initial impression in limited time, high processing demand automatically allocates more cognitive resources to recall schema-relevant information. This is because existing correlations produced by relevance, facilitate retrieval of information. Secondly, schematic knowledge improved recognition of schema-irrelevant information vs. schema-relevant information (Black et al., 1979; Elio and Anderson, 1981; Rojahn and Pettigrew, 1992; Lampinen et al., 2001), because schema-irrelevant objects are more distinctive (Rajaram, 1998) and likely to trigger conscious recollections (Lampinen et al., 2001). The cited studies showed that the relation between remembered information and the structure of schemata has a significant impact on memory performance.

When looking deeper into the relationships between memory and schemata to understand humans’ knowledge acquisition system, one question cannot be avoided: what is the structure of a schema? Sentis and Burnstein (1979) hypothesized that schema-relevant information is represented in memory as an integrated unit. They found that, when the number of to-be-recognized relationships increases, response time (RT) for answering questions regarding schema-relevant information remained stable, compared to schema-irrelevant information. Based on these findings, they proposed that schemata had an integrative function, which could organize schema-relevant information into the formation of highly integrated memory structures. Memory configuration is highly structured, rather than simply being a list of features or properties (Graesser and Nakamura, 1982). Studies on story schema revealed that a schema could be a sequence of causally related events (Schank and Abelson, 1977; Warren et al., 1979), a hierarchically interactive model (Rumelhart, 1977), a set of basic nodes in a tree structure (Mandler, 1978), or an embedded episode structure (Nezworski et al., 1982) which contained a causal relation between two episodes. To avoid the limitations of subjective reports, some indirect measures have been adopted for conducting experiments on schemata, including the comparison of recall distortions, with story structure set in advance, and analyzing between-subject memory performance (Stein and Glenn, 1975). Some experiments evidenced the existence of implicit knowledge structures with the dual purpose of differentiating relational invariances and configuring all structures (Reber and Lewis, 1977), supported by Read (1987). These demonstrated how the process model of knowledge structures (Schank and Abelson, 1977) could construct causal scenarios by relating actions in sequence. Ensar (2015) pointed out that a schema is a set of information clusters about well-connected spaces, events, people, and actions. The “restaurant schema,” for example, contains the action-eating information cluster (Eysenck and Keane, 2015). In summary, previous studies focused on the effects of schematic structure on the behavioral performance of a memory task, such as describing a learned story, to infer the structure and function of schemata. However, these methods used qualitative measures that were unable to reveal the schematic structure directly. Thus, a quantitative and endoscope-type tool is needed to gain more detailed knowledge on the structure of mental schemata.

In this study, we proposed the Reproduction of Categorical Knowledge (ROCK) tool, a novel method for uncovering the structure of mental schemata to explore the organization of implicit knowledge directly. The ROCK tool applies the serial reproduction paradigm and hierarchical clustering analysis to magnify memory bias and uncover inner configurations of fragmented information items, using the strength of association. We also conducted behavioral experiments to test the validity of the tool.

## The Rock Tool

The ROCK tool was designed to measure categorical structure in schemata. Categories form the primary structural features of schemata, with each category containing specific types of information and satisfying different information retrieval demands in the schemata (Trabasso et al., 1981). Srull and Wyer (1979) asked participants to select three of four words to form a sentence, demonstrating that the processing of the meanings of words activated the schematic categories to which those words belonged. This suggests that categories are the basic schematic units for the organization of knowledge.

The Supervised and Unsupervised Stratified Adaptive Incremental Network model (Love et al., 2004) was proposed to describe how categories worked in the assimilation of knowledge: old knowledge would be recruited into closely related categories, while for surprising events, a new representational cluster would be created, meaning that categories guided the process of encoding knowledge. Pecher et al. (2011) modified the definition of distribution of categories by redefining the relative distances of knowledge; they suggesting that same-category schematic knowledge is bounded in the same region (clustered together) compared to different-category knowledge. Because a schema is a pattern of thought or behaviors that organizes categories of information and the relationships among them (Brewer and Treyens, 1981; Taylor, 1981; Graesser and Nakamura, 1982), category-acquisition is the process of encoding schema-consistent items as unitary wholes and schema-inconsistent items as discrete propositions (Sentis and Burnstein, 1979). Considering the importance of category, we chose it as the focus of the current Experiment, aimed at developing a tool to uncover the categorical structure of knowledge and, more specifically, the category hierarchy describing multiple levels of items with within-category relations and between-category gaps.

The ROCK tool includes a measure procedure through behavioral experiments, and its corresponding data analysis. The core experimental method of this tool is the serial reproduction paradigm (Bartlett, 1932; Hall, 1950; Ost and Costall, 2002; McIntyre et al., 2004). In this paradigm, participants are required to reproduce the memorized stimuli (such as words), and these reproduced stimuli become the next participant’s memory stimuli. This process is performed for several times, such that the sequence of responses becomes the outcome of the serial reproduction process. In a classic example, Bartlett (1932) presented participants with a hieroglyph resembling an owl. The first participant memorized it and drew it from memory, and subsequent participants reproduced it from the previous participant’s drawing. The serial reproduction process resulted in last participant’s drawing to be transformed into a cat. As shown by Gelman et al. (2014), the interpersonal transmission process in the serial reproduction paradigm can be regarded as a Markov chain^1^. In such a chain, systematic memory bias will constantly accumulate, and even weak biases could be magnified to gradually emerge from noisy data (Suchow et al., 2017; Whalen and Griffiths, 2017). Some recent studies have reported the validity of the serial reproduction paradigm. For example, Uddenberg and Scholl (2018) revealed memory bias for faces through the serial reproduction paradigm. Participants viewed a briefly presented image of a face, which was picked from a smooth racial continuum, and reproduced the face for the next participant to memorize. The results showed a small reproduction bias favoring white, indicating the existence of a white face schema.

From a Bayesian perspective, memory biases revealed by serial reproduction reflect participants’ shared prior distribution about the memory stimuli (Xu and Griffiths, 2010). Since prior distribution represent the structure of people’s knowledge (Griffiths et al., 2010a,b; Tenenbaum et al., 2011), the memory biases revealed by serial reproduction can be perceived as the manifestations of mental schemata. Previous studies (e.g., Griffiths et al., 2010b; Langlois et al., 2021) have reported that the reproduced items will converge toward the prototype of each category, as the number of generations increase, among those with categorical structured knowledge. In other words, in the reproduction chain, items are gradually distributed according to the categorical structure, i.e., within-category items are clustered while between-category items are distinguished. Therefore, a cluster analysis of the converged items in the serial reproduction process should uncover the categorical structure of mental schemata.

Consequently, accumulated memory biases are analyzed using a clustering algorithm after the serial reproduction experiment. The ROCK tool specifically performs a hierarchical cluster analysis (Ward, 1963) on the last participant’s responses in each chain, which aims to minimize within-category differences and maximize between-category differences of the items reproduced in the previous generation. This type of analysis initially regards *n* reproduced items as *n* categories. Each time *n* categories are clustered into *n−1* categories, the sum of the deviation squared (*T*^2^) would increase and two categories would be clustered with the minimum growth of *T*^2^. The iteration of clustering categories would carry on unless the decisive index (*R*^2^) grows big enough. *T*^2^ and *S* are represented as the sum of between-category and within-category squared deviations, respectively, represented by following equations:

$$T^{2}=\sum_{j=1}^{n}{\left(x_{ij}-\bar{x_{i}}\right)}^{`}\left(x_{ij}-\bar{x_{i}}\right)$$

$$S_{k}=\sum_{i=1}^{k}\sum_{j=1}^{n_{i}}{\left(x_{ij}-\bar{x_{i}}\right)}^{`}\left(x_{ij}-\bar{x_{i}}\right)$$

where *n* is the number of reproduced items, *k* is the total number of categories, *n*_i_ is the number of items in the *i*-th category, $\bar{x_{i}}$ is the average of items in the *i*-th category, and *x*_ij_ is the *l*-th item in the *i*-th category.

*R*^2^ is the index deciding the final number of clustered categories, which is represented by:

$$R^{2}=1-\frac{S_{k}}{T^{2}}$$

An increasing *R*^2^ suggests that the distance between within-category items is shrinking and between-category items are becoming distinguished. If *R*^2^ grows big enough without a sudden increase, the human iteration could be terminated as this indicates that the reproduced items have been clustered into few enough categories and maintains stability, which has converged to the distribution of memorized information. This clustering analysis will reveal the organization and structure for all items, and is eventually presented in a hierarchical tree (Szekely and Rizzo, 2005). Thus, in theory, the hierarchical structure of mental schemata can be potentially unraveled by the ROCK tool. Therefore, we designed behavioral experiments to test the validity of the ROCK tool.

## Experiment 1

In order to test the ROCK tool, we chose stimuli with intrinsic structures as memory items for the serial reproduction procedure. Two kinds of stimuli with categorical structures were adopted, namely categorical stimuli with structures already acquired by the participants, and stimuli newly learned through a schema-acquisition process (De Brigard et al., 2017). Experiments 1a and 1b tested the ROCK tool by revealing participants’ pre-acquired knowledge structures. Personality traits were chosen as stimuli, as they are typical of knowledge with a well-defined categorical structure. If the ROCK tool is valid, the results should be consistent with participants’ mental theory of personality as shown in previous studies.

### Experiment 1a

We chose previously acquired knowledge of personality traits as stimuli to test the validity of the serial reproduction paradigm in revealing the structure of schemata. In Experiment 1a, stimuli were chosen from surface traits of personality (Fiske, 1949). Previous studies have found structural associations between personality descriptors (Tupes and Christal, 1961; Norman, 1963; Costa and McCrae, 1995; Shrout and Fiske, 1995; Bagby et al., 2005; Cattell and Mead, 2008), suggesting that people possess a shared inner personality theory. For instance, Norman (1963) discovered five orthogonal personality factors using peer nomination rating methods. Since the factor analysis approach was applied to personality studies, the categorical structure of personality traits was further clarified, resulting in a five-factor personality model (Digman, 1990) and the Big Five theory (Goldberg, 1993; Costa and McCrae, 1995). The Big Five Theory proposes five broad dimensions, named *openness*, *conscientiousness*, *extroversion*, *agreeableness*, and *neuroticism*, to describe and classify human personality traits. Thus, the Big Five theory can be considered as a mental schema, due to existing evidence indicating the utility of Big Five factors to guide memory and process social information (Smith and Kihlstrom, 1987; Edwards and Collins, 2008; Zhou et al., in press).

Based on the mechanism of the ROCK tool, we expected same-category personality traits in participants’ mental schemata to be easier to recognize, while cross-category personality traits would be relatively more likely to be ignored. During the interpersonal transmission chain, memory bias derived from the tendency to choose category-consistent traits to form a reconcilable and understandable personality would be magnified and ultimately converged to form several clusters of category-consistent traits. Thus, participants gradually develop memory biases that reflect their schematic structures during the behavioral experiment stage, and a cluster analysis of memory items can reveal the corresponding schema structure. Therefore, the ROCK tool can be considered valid to uncover the structure of mental schemata, if its clustering results in the present experiment are consistent with the categories of the Big Five personality theory.

All the measures and manipulations performed in Experiment 1 are reported below. No more data were collected contingent on initial analysis.

#### Method

##### Participants

According to Nahari et al. (2015), once a chain comprised more than four participants, reproduced information would converge. A recent study which applied the serial reproduction paradigm to personality trait recognition (Zhou et al., in press) also showed that stable convergence could be achieved for about seven participants in each chain. Thus, we allocated 10 chains with 10 participants to ensure convergence of results; 100 undergraduate students (47 females) were recruited from Zhejiang University, ranging from 18 to 24 years (Mean age = 20.8 years). Female and male participants were randomly allocated to different chains. All participants gave informed consent prior to participation and were paid or received course credit for their participation. This study (and all subsequent studies) was approved by the institutional review board at the Department of Psychology and Behavioral Sciences, Zhejiang University.

##### Stimuli

We chose 44 surface trait words from Fiske (1949), forming 22 groups of words with each group containing a word related to a positive trait (e.g., “trustful”) and another related to a negative trait (e.g., “suspicious”). These words were translated into four-character Chinese words in a standardized form, used in previous studies (Luo and Dai, 2015). Thirty-six standardized facial images (18 per gender) were used as ID photos, and names were generated for each photo using random combinations of the most commonly used Chinese surnames and first names. The photos were evaluated by the selection criteria of few facial features and low attractiveness. In order to avoid the possible influence of additional social information on the experimental results, only the part of the photo above the shirt collar was kept. The mean age of the people in the photos was 20.4 years (SD = 1.2).

##### Procedure

**FIGURE 1:**
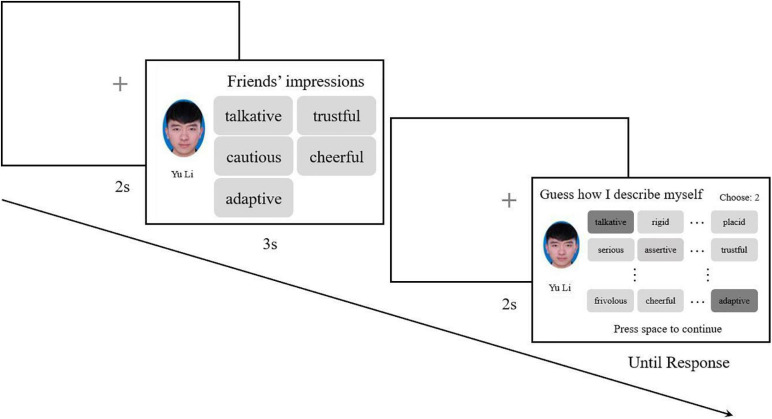
For the first participants of each chain, five randomly chosen, non-repetitive words were combined with a random facial image for one trial, and each face would appear only once. In the probe phase, 44 words were listed in a 11 × 4 matrix for participants to choose from; the order of the words would be randomized for each trial. The combination of facial images and five impression words recognized by the previous participants would be randomly presented to subsequent participants.

The participants were randomly assigned to 10 chains of 10 participants each. Participants were told that in each trial they would view an ID photo of a person with five impression (personality trait) words used to describe the person by their friends. Participants needed to memorize all the information presented and pick out the memorized impression words from a 44-word list in the subsequent probe phase. Additionally, participants were informed that if any word was forgotten, they could guess and choose the most likely word. Every participant completed 36 trials. Words picked out by one participant would become the next participant’s memorized materials. For each chain, the next participant would not start their experiment unless the previous participant had finished the whole experiment (Figure 1).

#### Data Analysis

In each chain of 10 participants, memory bias continually accumulated as the information spread. As a result, the final output from the last participant contained the most magnified bias effect caused by all 10 participants’ prior knowledge of personality traits. Thus, we chose the last participant’s answer of each chain, and used co-word analysis (Hamers et al., 1989; Callon et al., 1991; Ding et al., 2001; Xianzi et al., 2014) to assess the association strength between two different word groups by calculating their co-occurrence. The parameter Salton Index was used in this method. The parameter Salton Index of association strength between word i and word j is calculated using the following equation:

$$Saltonindex\left(i,j\right)=\frac{C_{ij}}{\sqrt{c_{i}c_{j}}},0<Saltonindex<1$$

In our Experiment, c_ij_ was the number of co-occurrences in word group i and word group j. For example, consider the word groups *conscientious* – *not conscientious* (group i) and *trustful* – *suspicious* (group j). If *conscientious* (in group i) and *trustful* (in group j) are simultaneously used to describe the same facial image by the 10th participant in a chain, then we can say that the word group i and the word group j co-occur once, and c_ij_ is defined as the total number of such co-occurrences in a participant’s recognition results. Further, c_i_ was the number of times that word group i occurred (any one word in the group) to describe any facial image in the 10th participant’s answers in each chain, while *c_j* was the number of times that word group j occurred in the 10th participant’s answers. The Salton indices between all word groups were calculated separately based on the recognition results of 10 participants (i.e., the last participant in each chain) and then averaged.

A 22 × 22 matrix of Salton indexes was created to represent the association strength between two different word groups. The matrix was then transformed into a dissimilarity matrix (De Soete, 1984; Day, 1987; Bezdek et al., 2007), and the parameter dissimilarity index was given by the following expression:

$$dissimilarityindex\left(i,j\right)=1-\frac{C_{ij}}{\sqrt{c_{i}c_{j}}},0<dissimilarityindex<1$$

The dissimilarity index represented the relatedness of two-word groups; if it was close to zero, the groups were related. Based on the dissimilarity matrix, hierarchical cluster analysis was conducted to reveal the categorical hierarchy of the reproduced words, reflecting the schema structure of participants’ internal theory of personality. Ward (1963) method with chi-square measure was used in the hierarchical cluster analysis.

#### Results

**FIGURE 2:**
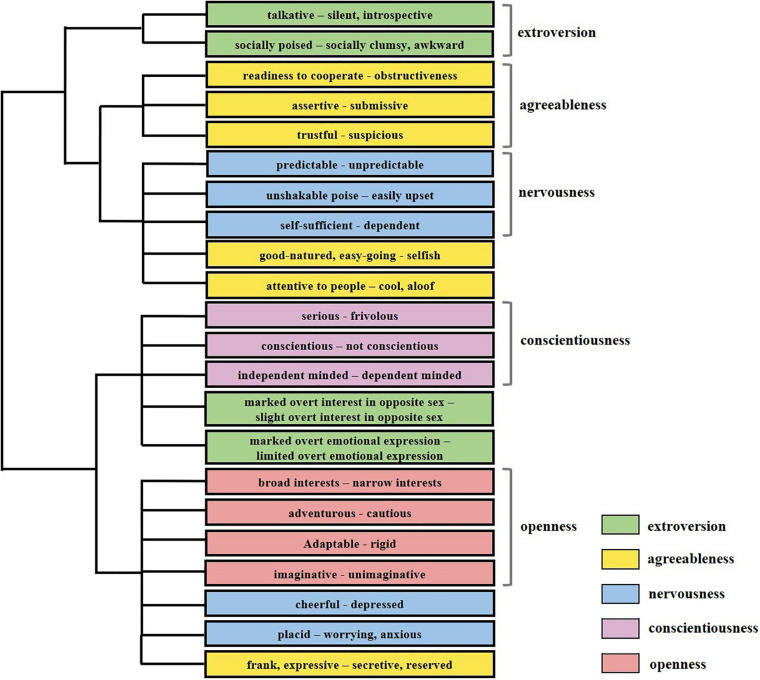
Apart from fifteen word groups clustered into correct dimensions, seven word groups were clustered into incorrect dimensions compared with results we identified. The pairs *attentive to people* – *cool, aloof; good-natured*, *easy-going* – *selfish*, and *frank*, *expressive* – *secretive, reserved* were not clustered into agreeableness. *Marked overt interest in opposite sex* – *slight overt interest in opposite sex;* and *marked overt emotional expression* – *limited overt emotional expression* were not clustered into extroversion. *Cheerful* – *depressed*; and *placid* – *worrying, anxious* were not clustered into neuroticism. Colors denotes word groups’ categories in the Big Five personality theory.

The analysis revealed five personality dimensions: extroversion, agreeableness, neuroticism, conscientiousness, and openness. The dendrogram of the cluster analysis is shown in Figure 2. The cluster analysis revealed a similar structure to the Big Five theory (McCrae and Costa, 1992). The number of identified trait words was categorically allocated to each dimension and the word groups distinguished in the experiment generally fit the Big Five theory of personality; for example, *broad interests* – *narrow interests* was clustered into the dimension *openness*. Cluster names that we identified were given for each cluster, as shown in Table 1.

**TABLE 1:** Five clusters of personality trait words.

No.	Cluster name	Members of cluster
1	Extroversion	Socially poised – socially clumsy, awkward; talkative – introspective
2	Agreeableness	Readiness to cooperate – obstructiveness; assertive – submissive; suspicious – trustful
3	Neuroticism	Predictable – unpredictable (day-to-day behaviors and attitudes); easily upset – unshakable poise; dependent – self-sufficient
4	Conscientiousness	Frivolous – serious; conscientious – not conscientious; dependent-minded – independent-minded
5	Openness	Broad interests – narrow interests; cautious – adventurous; rigid – adaptable; imaginative – unimaginative

We used Goodman and Kruskal’s (1963) lambda (λ) to measure the association between the categories of the Big Five theory and the ROCK tool, and the results reported a significant association (λ = 0.548, *p* = 0.003).

#### Discussion

Results of Experiment 1a showed the five dimensions of the Big Five theory emerging from the final output of the serial reproduction procedure. Fifteen of the word groups were clustered into correct dimensions, although some were clustered into a dimension different from McCrae and Costa’s (1992) model. For example, *assertive* – *submissive* (here, clustered into *agreeableness*) could also be clustered into *openness* in their model. Some word groups not included in McCrae and Costa’s (1992) model were clustered into dimensions containing semantically similar words, for instance, *frivolous* – *serious*, which was distinguished in our results, was similar to *thorough* – *careless* in McCrae and Costa’s (1992) model.

A plausible reason for this minute difference between the Big Five personality structure and the revealed hierarchy is that the Big Five theory may not be completely consistent with people’s schemata of personality (Smith and Kihlstrom, 1987; Dabady et al., 1999). For instance, Smith and Kihlstrom (1987) demonstrated that the factors and traits in the Big Five dimensions were not discriminatory, and there were some overlaps in the subordinate traits. Therefore, this small deviation of the current clustering results from the Big Five theory is relatively tolerable.

In summary, the ROCK tool revealed the structure of the perceived personality of others, which was generally consistent with the Big Five theory of personality.

### Experiment 1b

Experiment 1a revealed the structure of personality at a higher level through the serial reproduction paradigm. According to the Big Five theory (Goldberg, 1993; Costa and McCrae, 1995), each personality dimension has its own sub-dimensions. For Experiment 1b we chose *extroversion*, one dimension of the Big Five traits, to replicate the results of Experiment 1a at a lower level of personality structure. Extroversion traits have six sub-dimensions: *enthusiasm*, *prosociality*, *energy*, *arbitrariness*, *sensation-seeking*, and *positive emotion* (Costa and McCrae, 1995; Luo and Dai, 2015). We chose the extroversion dimension due to the high reliability of all its sub-dimensions within the Chinese context (Luo and Dai, 2015), while the other dimensions report relatively lower reliability (Cronbach’s α < 0.7) in their sub-dimensions. Therefore, extroversion is a more reliable and stable dimension for this experiment.

#### Method

##### Participants

One hundred (10 chains with 10 participants each) undergraduate students were recruited from Zhejiang University, ranging from18 to 26 years (Mean age = 21.9 years). Female and male participants were randomly allocated to different chains. Those who had participated in Experiment 1a were excluded. All participants gave informed consent prior to the experiment. They were paid or received course credit for their participation.

##### Stimuli

Twenty-one extroversion words were chosen from Pervin and John (1999), translated by Huang (2003). As the number of positive and negative words were not balanced (15 positive words and six negative words), we selected an additional 15 words (three positive and 12 negative) used in previous personality studies (Luo and Dai, 2015) to create a word set containing 18 positive words and 18 negative words; all 36 were four-character Chinese words. The 36 facial images and corresponding names used in Experiment 1a were adopted for Experiment 1b.

##### Procedure and data analysis

**FIGURE 3:**
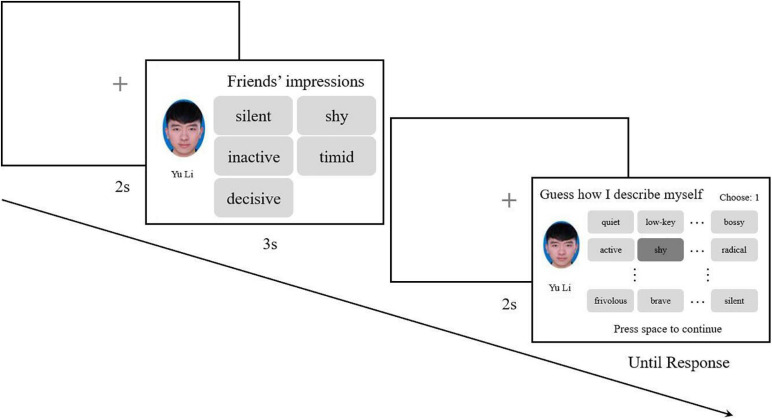
For the first participants of each chain, five non-repetitive words were randomly chosen and combined with a randomly chosen facial image in one trial, each face appearing only once. In the probe phase, 36 words were listed in a 9 × 4 matrix for participants to choose from. In the probe phase of each trial, the presentation of the 36 words would be randomized. For subsequent participants, the combination of facial images and five impression words reproduced by the previous participants would be presented as the items to memorize.

The procedure (Figure 3) and data analysis of Experiment 1b was identical to that of Experiment 1a.

#### Results

**FIGURE 4:**
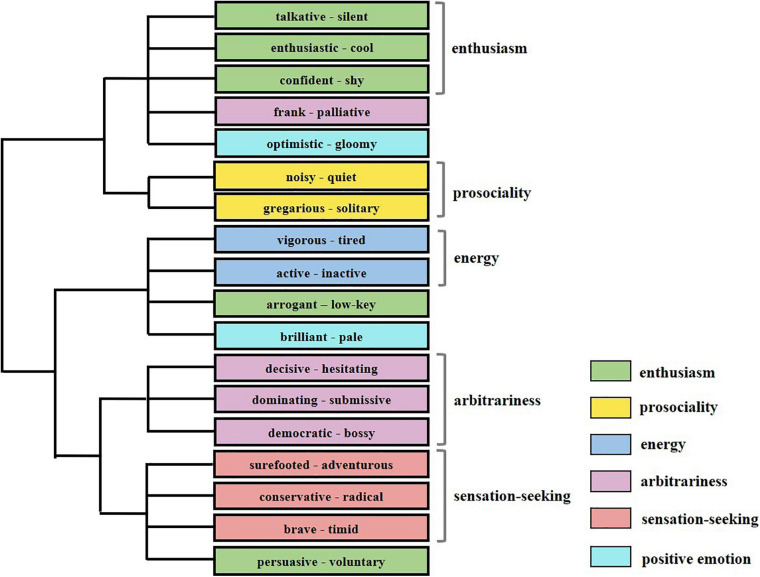
The word group *frank – palliative* was not clustered into *arbitrariness*. The pairs *optimistic – gloomy* and *brilliant – pale* were not clustered into positive emotion, which was not distinguished in the results. *Arrogant – low-key* was not clustered into enthusiasm. Colors denotes word groups’ categories in the Big Five personality theory.

Five dimensions were revealed by the analysis, while one dimension (positive emotion) failed to emerge. Thirty words groups were clustered into correct dimensions while five-word groups (a pair of positive and negative words) failed. The dendrogram of the cluster analysis is shown in Figure 4. Cluster analysis revealed a very similar structure to the structure of extroversion, and 75% of word groups from the dimensions of *enthusiasm*, *arbitrariness*, and *sensation-seeking* were distinguished in the experiment, consistent with Luo and Dai’s (2015) findings. For the dimensions *prosociality* and *energy*, 50% of word groups were distinguished. Identified cluster names were given for each cluster as shown in Table 2. There was also a significant association (λ = 0.654, *p* < 0.001) between the classifications obtained by the Big Five theory and the ROCK tool.

**TABLE 2:** Five clusters of extroversion words.

No.	Cluster name	Members of cluster
1	Enthusiasm	Talkative – silent; enthusiastic – cool; confident – shy
2	Prosociality^1^	Quiet – noisy; gregarious – solitary
3	Energy	Vigorous – tired; active – inactive
4	Arbitrariness	Decisive – hesitating; dominating – submissive; democratic – bossy
5	Sensation-seeking	Adventurous – surefooted; radical – conservative; brave – timid

*^1^In this article, being close to social groups was defined as prosociality here.*

#### Discussion

In Experiment 1b five dimensions were distinguished while one dimension (positive emotion) failed to be revealed, in line with Luo and Dai’s (2015) personality model. The Chinese version of *brilliant – pale* had at least two meanings from different perspectives (describing someone’s face or describing their character), which may have caused ambiguity. Only two out of 18 words groups belonged to *positive emotion*, suggesting that this dimension was difficult to distinguish. In total, 13 words groups were clustered into their correct dimensions. This suggests that the serial reproduction paradigm can broadly reveal the structure of extroversion.

Consistent with Experiment 1a, the results of this experiment could be affected by the differences between the Big Five theory and people’s schemata about personality. Therefore, a more clearly structured mental schema that can be manipulate in an experiment should be employed to assess the validity of the ROCK tool better.

## Experiment 2

Combining Experiment 1a and Experiment 1b, our results show that the serial reproduction paradigm is an effective tool for exploring the structure of mental schemata. However, a limitation for the familiar stimuli (personality traits) used in these experiments is that we do not possess a precise picture of their structure. Furthermore, the consistency of Big Five personality theory with the mental schemata of personality traits is still a controversial topic (Smith and Kihlstrom, 1987). Therefore, in Experiment 2, to verify the validity of the ROCK tool further, we used stimuli with a whose structure was artificially generated structure. Compared to personality traits that can be seen as a “rough answer,” the structure of these artificial stimuli is determined by a generative model, and thus, it can be considered a “standard answer” and used as a more unambiguous test of the ROCK tool.

Participants can acquire the structure of these stimuli through a stage of implicit-learning and develop a temporary cognitive schema. In the following experiment, we design the implicit-learning stage as a modified two-back task (Kirchner, 1958), in which the stimuli sequence was generated according to their inner structure defined by the generative model. Participants could implicitly learn the stimuli structure through this task (as confirmed in Experiment 2a). The serial reproduction paradigm was then performed to test whether the structure participants acquired through the implicit learning procedure could be revealed using the ROCK tool (Experiment 2b). If the tool was valid, the result would be consistent with the inner structure of the presented stimuli. All measures, manipulations, and exclusions in Experiment 2 are reported below. No more data were collected contingent on initial analysis.

### Experiment 2a

#### Methods

##### Participants

Eighteen undergraduate students (nine females) were recruited from Zhejiang University, ranging from19 to 24 years (Mean age = 21.4 years). A sensitivity power analysis indicated that for a paired-samples *t*-test (*N* = 18, α = 0.05), with a statistical power of 1 – β = 0.80, the minimum effect size *w* would be 0.80; for independent *t* test, *N* = 18, α = 0.05, and a statistical power of 1 – β = 0.80, the minimum effect size *d* would be 1.72 (Faul et al., 2007). All participants provided informed consent and were paid or received course credit for their participation.

##### Stimuli

**FIGURE 5:**
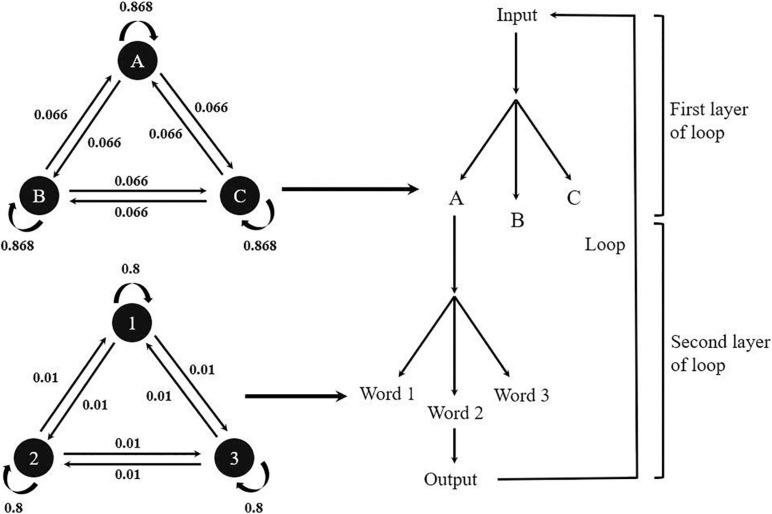
Both Markov chains were developed with a larger possibility of transferring to itself and a smaller possibility of transferring to other categories or words. In the first trial, the choice of categories and words were uniform. In the (*t*)th trial, the choice was decided by the previous chosen categories and words in the (*t*–1)th trial. For example, if Category A was chosen in (*t*–1)th trial, then in the (*t*)th trial, there was an 86.8% chance of picking Category A again and an 8.68% chance of picking either Category B or C. If the category choice was different from the Category A, the chosen probability of words in the second layer of Category B or C is 1/3; if the category chosen was identical, the chosen possibility of words in the second layer was decided by the previous one chosen word. For example, for the second loop, if Category A was chosen again, and Word 1 was picked in (*t*–1)th trial, there was an 80% chance of picking Word 1 again and a 10% chance of picking either Words 2 or 3. The transfer probability of the Markov models was decided to confirm that the word in (*t*)th trial had a 50% chance to replicate the word in the (*t* + 2)th trial.

Nine words that were not semantically related were chosen as the stimuli for Experiment 2a and were displayed as two-character Chinese words. An artificial tree structure containing three branches was created to generate the presenting sequence (presented in the two-back task) of these words. Each branch contained three words and their occurrence was determined by a Markov chain model (Nummelin and Tuominen, 1982). In this probabilistic model, the generative process of each word contained two cyclical Markov chains. One of the Markov chains decided the occurrence of tree branches, and the other decided the occurrence of words. Due to the properties of the Markov chain, the occurrence of each word was decided by the previous chosen word. As shown in Figure 5, the generative model ensured that the words under the same branch – or from the same category – were more likely to occur at an adjacent position in the presenting sequence, with a relatively low probability of “jumping” to another branch. In this way, the structure (i.e., the hierarchical tree) of words would be reflected by the word sequence.

The nine words used in Experiment 2 and their structure are shown in Table 3.

**TABLE 3:** Words used in Experiment 2.

Category	Members of cluster
A	Sky, car, and swimming
B	Celery, shanghai, and number
C	Patch, piano, and cell

##### Procedure

**FIGURE 6:**
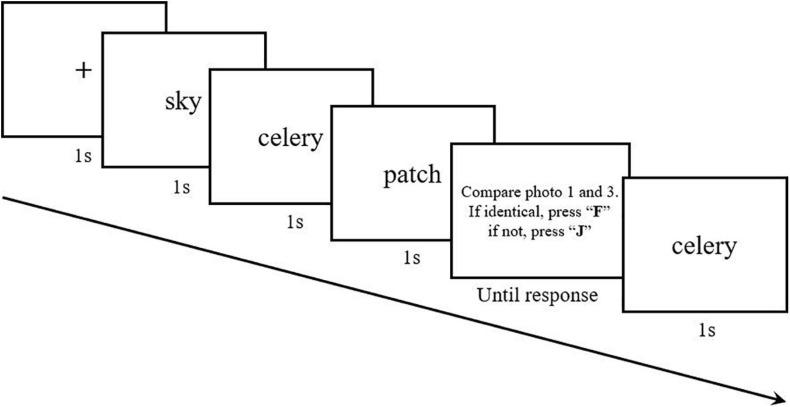
Each trial contained five words and participants needed to compare the current word and the one appearing before the previous word. This meant they needed to start comparison from the third word. Once the third word disappeared, the judging phase started and participants had to press the keyboard to indicate their judgment. There was no feedback. After judging, the fourth word appeared.

Experiment 2 comprised two sub-experiments. Prior to the main experiment (Experiment 2b), we needed to confirm that participants were able to learn the structure implicitly from the word sequence presented in the modified two-back task. In our experiment, the two-back task carried the word sequence, forcing participants to attend and memorize the stimuli with their inner structure. Thus, Experiment 2a tested the effect of implicit learning through the two-back task, to verify that the structure of artificial stimuli could be implicitly acquired. All participants were tested individually and were not informed of the inner structure of word sequences presented in the experiment. The whole task contained two parts. In the first part (learning phase), participants were told to compare the current word and the one appearing before the previous word (i.e., two-back task). Participants were required to react as quickly and accurately as possible. If the word presented was identical to the word before the previous one, they had to press F; if not, they had to press J (Figure 6). The experiment contained three blocks of 70 trials each. Each trial contained five words, presented in the middle of the screen.

In the second part (testing phase), the instruction was identical to the first part, with 13 blocks of 30 trials each. We implemented breaks after each block in each of the two experiments, where participants could pause and press the spacebar to start the next block.

For the first part, three blocks would start from any of the branches in the Markov chain model. For example, if one block started from Branch A, the remaining blocks would start from Branches B and C, respectively. All trials in one block would start from the corresponding branch. The sequence of the three blocks was random. For the second part, similar to the manipulations of Weiermann et al. (2010), we created regular stimuli according to the pre-defined structure. In the normal phase, Blocks 1–7 also followed the rules defined by the Markov chain model. Each trial in Blocks 1–7 would randomly start from any of branches in the model. After this phase, we administered one test to assess the effects of implicit learning. The random phase (Blocks 8–13) consisted of two regular blocks (8 and 9) like Blocks 1–7, two random blocks (10 and 11), and again two regular blocks (12 and 13). Compared with regular blocks, random blocks contained stimuli which was picked out randomly from the stimuli of nine photos with words. After 13 blocks had been finished, in order to assess their explicit knowledge of the presented word sequences, participants were asked to rewrite the word sequence on a paper sheet. They were allowed to write down any possible word sequences and guess if necessary. We calculated the number of correct sequences for each participant, in which all words came from the same branch of Markov chain model.

#### Data Analysis

In order to assess the effect of implicit learning, we analyzed RTs of several key blocks with a paired-samples *t*-test in the second part of Experiment 2a, including mean RTs of Blocks 1 and 7 and dropping score (mean RTs differences of the random Blocks 10 and 11 and the adjacent regular Blocks 8, 9, 12, and 13). Additionally, to assess the effect of explicit learning, we calculated the mean number of sequences answered correctly for all participants. For those who answered correct sequences at a higher than average level (suspiciously acquired explicit knowledge), we used an independent-samples *t*-test to analyze the dropping score of the explicit-learning group and the implicit-learning group.

#### Results and Discussion

**FIGURE 7:**
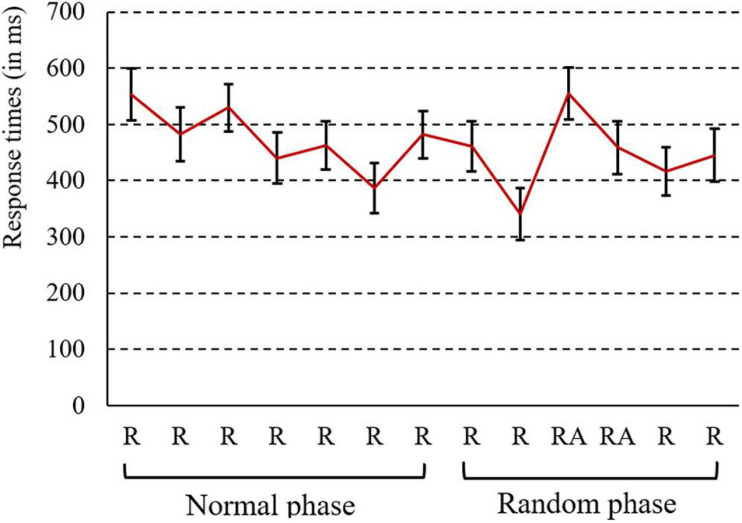
Response time results for the second part of Experiment 2a (R = regular block; RA = random block). Error bars show standard errors.

The results of RT are shown in Figure 7. There was a dropping trend in RTs in Blocks 1–7, which indicates the existence of the effect of learning. The normal score (mean RT of Block 1 minus that of Block 7) was 70 ms (SE = 17), and the RTs in Blocks 1 and 7 were significantly different with a paired-samples *t*-test [*t*(17) = 3.9, *p* < 0.005, *d* = 0.38]. In the random phase, implicit learning was revealed. The dropping score (mean RTs of Blocks 10 and 11 minus that of Blocks 8, 9, 12, and 13) of all participants was 93 ms (SE = 7). RTs were significantly slower in random blocks compared to regular blocks with a paired-samples *t*-test [*t*(17) = 12.9, *p* < 0.001, *d* = 0.51], indicating that participants would react slowly when they encountered a random block. This suggests that participants did learn the structure of words, therefore facilitating performance in the two-back task.

In addition to implicit learning, the structure of words was partly learned explicitly. The proportion of reporting correct word sequences was 1.28 out of 3 (SD = 0.73). If participants correctly distinguished more than two correct word sequences (in other words, two branches of the Markov chain model), they were suspicious of learning the structure in an explicit way. A total of six participants were identified to be explicitly acquiring individuals; five correctly scored 2 and one scored 3.

To separate the effects of explicit and implicit learning, we compared the RTs of the explicit-learning group (six participants) and the implicit-learning group (12 participants). The mean dropping score was 94 ms (SE = 10) for the explicit-learning group and 92 ms (SE = 9) for the implicit-learning group. These two groups did not differ in dropping score with an independent-sample *t*-test (*p* = 0.873). For the implicit-learning group, RTs of Blocks 10–11 differed significantly from that of Blocks 8, 9, 12, and 13 with a paired-samples *t*-test [*t*(11) = 9.6, *p* < 0.001, *d* = 0.60]. These results show that even for explicit-learning participants, explicit knowledge brought little extra benefit for their behavioral performance.

In the two-back experiment, participants indeed implicitly learned the structure produced by the Markov chain model. Thus, the stimuli and learning procedure could be used to test the validity of our ROCK tool for revealing what participants implicitly learned, in Experiment 2b.

### Experiment 2b

#### Methods

##### Participants

One hundred (10 chains with 10 participants each) undergraduate students (40 females) were recruited from Zhejiang University, ranging from19 to 24 years (Mean age = 21.4 years). Female and male participants were randomly allocated to different chains. Participants who had been involved in Experiments 1a and 1b were excluded. All participants gave informed consent and they were paid or received course credit for their participation.

##### Stimuli

The stimuli were identical to that in Experiment 2a.

##### Procedure

**FIGURE 8:**
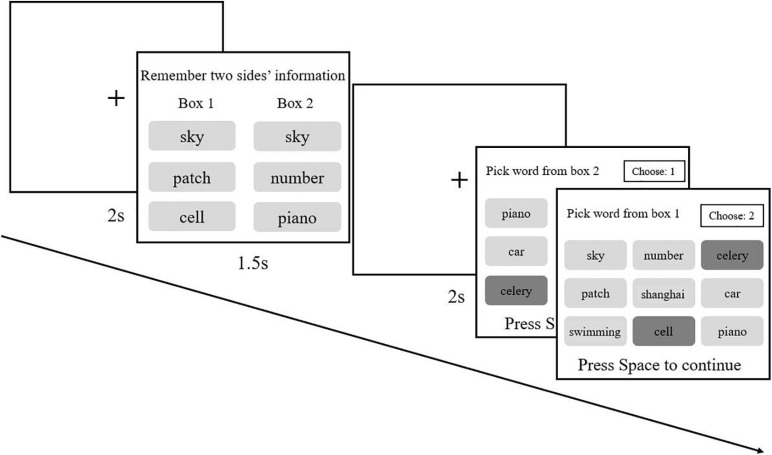
For the first participants of each chain, two groups of three non-repetitive words were randomly chosen and appeared on two sides. In the probe phase, nine words were listed in a 3 × 3 matrix for participants to choose from with a random detecting sequence for two boxes. For example, participants might be told to choose three words they remembered for box 2, and once they had picked three words and pressed the spacebar to confirm, another probe phase of the box 1 appeared. In the probe phase of each trial, the presented nine words would be randomized. For subsequent participants, three words from both boxes recognized by the previous participants would be presented randomly.

In Experiment 2b, participants were asked to perform the modified two-back task (learning), identical to that used in Experiment 2a. After that, all of them entered the serial reproduction procedure. They were instructed that 18 trials were to performed with two boxes, and each box contained three words, which appeared on both sides of the screen. Participants needed to memorize all the words in the two boxes. After waiting for a while, they had to select three memorized words from a probe word list containing nine words. Words picked out by the previous participant would become the next participant’s memorized materials. For each chain, the next participant would not start their experiment unless the previous participant had finished the whole experiment. Additionally, participants were informed that if any word was forgotten, they could guess and choose the most likely word. The whole experiment contained 18 trials (Figure 8).

#### Data Analysis

The data analysis of Experiment 2b was identical to that of Experiments 1a and 1b.

#### Results and Discussion

**FIGURE 9:**
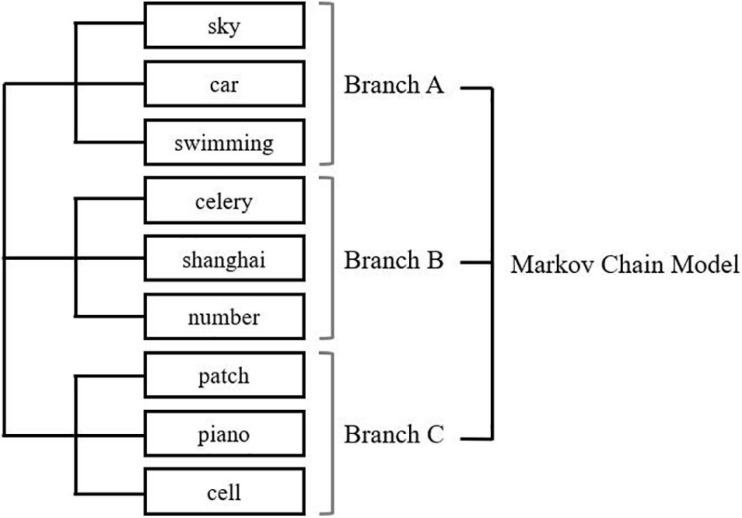
Cluster analysis results.

The structure revealed by the ROCK tool, shown in Figure 9, was identical to the structure we designed in the Markov chain model. In summary, interpersonal transmission in the reproduced chain magnified the memory bias toward schemata. Thus, the serial reproduction paradigm could reveal the structure of unfamiliar artificial stimuli. This experiment used more explicitly structured mental schemata and reported higher consistency between the revealed structure and the structure of learned schemata than Experiments 1a and 1b, which further emphasizes the validity of the ROCK tool.

## General Discussion

This study introduced a tool to reveal the structure of schemata, and demonstrated that it works, for both familiar and artificial stimuli. We used the serial reproduction paradigm to measure and magnify participants’ memory bias, which reflects the structural features of schemata in the memory task. Based on the magnified bias, a cluster analysis was conducted to reveal the categorical structure of mental schemata. In particular, the co-occurrence frequencies of the words at the end of the reproduction chain were used to calculate the association strength of the connections among memorized words. This formed the basis for a hierarchical cluster analysis performed to distinguish mental categories, thereby revealing the structure of schemata that participants had previously acquired or newly learned.

We tested the validity of the ROCK tool in revealing schematic structure through two major experiments. The results of Experiment 1a showed that the ROCK tool could reveal the structure of participants’ schemata for personality, which was generally consistent with the structure of the Big Five theory of personality (McCrae and Costa, 1992). Experiment 1b further tested the tool at a lower-level dimension of the Big Five traits, namely extroversion. The result showed that, for the dimension of extroversion, the structure uncovered by the ROCK tool was mostly consistent with the structure of extroversion (Luo and Dai, 2015). In addition to the familiar stimuli (i.e., personality traits) of Experiment 1, Experiment 2 tested whether the structure of knowledge that had been newly acquired could be revealed by this tool. Experiment 2 used a two-back paradigm to present structured word sequences for participants to learn, and then used the ROCK tool to reveal the structure of word sequences that participants had learned, testing whether the revealed structure was identical to the designed structure. Compared to Experiments 1a and 1b, we achieved a better result in Experiment 2b: the revealed structure was identical to the structure of Markov chain model. This might be due to a number of reasons. Firstly, the structure of stimuli in Experiment 1 were more complex than in Experiment 2 (five or six dimensions vs. three dimensions or branches); further, the designed structure in Experiment 2 contained only three words in each dimension, while in Experiment 1, there were at least three pairs of words (one positive and one negative). Larger complexity of the structure led to more difficulties in converging to “correct” clusters. Secondly, the personal definitions of personality words varied among participants, as did their own theories of personality. This introduces extra errors to the converging process in Experiment 1. Thirdly and most importantly, in Experiments 1a and 1b we tested participants’ perception of personality, the boundaries of which are relatively blurred because participants’ inner personality theories may not be fully consistent with the Big Five theory. In contrast, for the pre-defined model in Experiment 2, within-category distances were specially narrowed and between-category differences were magnified through setting a large transfer probability within categories and a small one between categories, leading to a more clear and explicit structure. This enabled participants to develop stronger schemata and show more stable memory biases. Therefore, these arguments taken together suggest that the ROCK tool revealed the schematic structure more easily in Experiment 2.

The serial reproduction paradigm has been widely used in exploring cultural stereotypes (Kashima, 2000; Lyons and Kashima, 2003; Kashima and Yeung, 2010), conflict arising from communication biases (Lee et al., 2014), social representation of concepts (Bangerter, 2000) and memory bias (Ost and Costall, 2002; Xu and Griffiths, 2010; Uddenberg and Scholl, 2018). The reproduced chain had been verified to unravel and magnify mental representations of different objects, scenes and causality of related items. Inspired by this work, we used it to reveal the structure of schemata. Similar ideas can been found in the work of Nahari et al. (2015), who explored recalling of traumatic events using the serial reproduction paradigm, and used trend analysis (broken line) to reveal qualitative memory bias. The ROCK tool brings memory bias into quantitative analysis. Additionally, the cluster analysis based on the co-word matrix could reveal the structure of words (i.e., the mental connections of words in schema) presented in our experiment. Similar methods have been widely used in the processing of abundant data to extract relations among items (Tijssen and Van Raan, 1989; Naptali et al., 2008; Liu et al., 2011; Xianzi et al., 2014; Yan et al., 2015), which could clearly exhibit the between-category differences and within-category relations.

Our ROCK tool has several advantages. First, it has been endowed with anti-noise ability; thus, continuous iterations among participants could weaken the effect of uncertain factors, and the magnified memory bias could dominate analyzed results. Second, this tool could be widely used for various kinds of stimuli, as long as the stimuli can be memorized and reproduced. Third, with the memory experiment as a cover, the ROCK tool can implicitly measure schemata as participants are unlikely to realize the true purpose of the experiment. Thus, the confusion caused by high-level cognition (such as using a strategy to finish the task according to the guessed purpose of the experiment) can be avoided.

Certainly, the ROCK tool has its limitations. Since serial reproduction requires participants to reproduce stimuli they had learned or memorized, the stimuli should be reproducible and relatively stable during interpersonal transmission. Otherwise, the stimuli would decay rapidly in the stimuli-memory-stimuli loop. Some materials, such as subjective feeling, procedural knowledge, and overwhelming information with a large amount of details, are inappropriate for reproduction. For instance, a text story that is too long for participants to remember in detail will rapidly lose its length in a few iterations of transmission, leaving little information for analysis. Another limitation is that, the schematic structure to be measured should be relatively constant across participants, so that it could generate a system bias that can be accumulated during the reproduction chain. Material with significant individual differences (e.g., the culture differences of value) is unlikely to converge in a reproduction chain.

Our results confirmed that schemata are highly structured. Structure aids our process of thinking, and allows information related to that which we are processing to converge and guide our action, decision-making, emotions, and neurological activities. Our results show that schema-related information was easier to recall (Deese, 1961). In the early phase of our reproduced chain (participants 1–5), presented stimuli were not consistent with participants’ schemata. In the condition with limited time to memorize, participants frequently made mistakes. The forgotten words would be replaced by those matching internal schemata, which is easy to access during retrieval. In the later phase of the reproduction chain, with systematic bias toward participants’ schema, stimuli were easier to memorize and retrieve. Cluster analyses of studies 1 and 2 clearly show that related stimuli would be grouped together to form a hierarchical structure, consistent with Sentis and Burnstein’s (1979) findings that schemata are structured to keep related information accessible, while unrelated information is differentiated in a hierarchical manner.

As for proactively acquiring schema in Experiments 2a and 2b, we introduced category learning as a means of acquiring structured knowledge. It has been used in neuroimaging studies (Patalano et al., 2001; Grill-Spector et al., 2006; Gureckis et al., 2011) and knowledge acquisition studies (Bower and Gilligan, 1979; Palmeri and Nosofsky, 1995; Sakamoto et al., 2004). According to earlier studies of category learning, this was supposed to be equivalent to schema acquisition (Love et al., 2004). Previous studies focused on category learning for acquiring structured knowledge in memory experiments (Davis et al., 2014; De Brigard et al., 2017), and findings showed the effect of schema on recognition memory with familiar stimuli (geometric figure and words) and unfamiliar stimuli (flowers generated by computers). These manipulations of experimental stimuli guided us to combine category learning and the ROCK tool to test the likelihood of directly unraveling schema.

In conclusion, our results have shown the potential of the ROCK tool for the exploration of schemata with categorical structure. The tool could be productively used in studies of schema acquisition and schema revelation.

## Data Availability Statement

The datasets presented in this study can be found in online repositories. The names of the repository/repositories and accession number(s) can be found below: https://osf.io/yzf5j/?view_only=7585a3f97d074d748cc918fd94b7b0c2.

## Ethics Statement

The studies involving human participants were reviewed and approved by the Ethics Committee of Zhejiang University. The patients/participants provided their written informed consent to participate in this study.

## Author Contributions

JZ and HC developed the study concept. BS and ZJ performed the experiments and analyzed the data. BS contributed to implement the tool and computational model. ZJ and JZ drafted the manuscript. All authors have approved the final version of the manuscript for submission.

## Conflict of Interest

The authors declare that the research was conducted in the absence of any commercial or financial relationships that could be construed as a potential conflict of interest.

## Footnotes

1 A Markov chain is a sequence of possible events in which the probability of each event depends only on the state attained in the previous event (Gagniuc, 2017).
